# Attention-Deficit/Hyperactivity Disorder With Family Relational Challenges and Persistent Gender-Nonconforming Expression in a Preadolescent Girl: A Case Report

**DOI:** 10.7759/cureus.117124

**Published:** 2026-09-29

**Authors:** Jonathan Bazile, Nadia Bernard, Ranwinder Kaur, Midhun Kumar Maganti, Kandathodi H Faizal

**Affiliations:** 1 Obstetrics and Gynecology, State University of Haiti, Port-au-Prince, HTI; 2 Family Medicine, Care Promotion PC, Canton, USA; 3 Internal Medicine, Sri Guru Ram Das University of Health Sciences, Amritsar, IND; 4 Neurology, Guntur Medical College, Vijayawada, IND; 5 Internal Medicine, Yigatti Healthcare, Pannicode, IND

**Keywords:** attention-deficit/hyperactivity disorder, behavioral parent training, family relational challenges, family therapy, gender-nonconforming expression, preadolescent girl

## Abstract

Attention-deficit/hyperactivity disorder (ADHD) in girls may be overlooked or diagnosed late because symptoms can be influenced by emotional dysregulation, compensatory behavior, and family or social context. We describe a 10-year-old girl who was referred for impaired concentration, impulsivity, academic disruption, and aggressive peer interactions. A comprehensive behavioral assessment supported a diagnosis of ADHD, combined presentation. Clinical evaluation also identified substantial conflict involving her parents, twin sister, and grandmother, together with a longstanding pattern of gender-nonconforming expression. Treatment consisted of individual psychotherapy, behavioral parent training, school accommodations, and family-based interventions targeting emotional regulation, executive functioning, and relational conflict. After six months, caregivers and teachers reported fewer aggressive incidents, improved classroom participation, and better emotional regulation, although attentional difficulties persisted. This case illustrates the importance of evaluating ADHD within the child’s developmental, educational, and family contexts while distinguishing gender expression from gender identity. Individualized, nonjudgmental, and developmentally appropriate care can address neurodevelopmental impairment without pathologizing a child’s personal expression.

## Introduction

Attention-deficit/hyperactivity disorder (ADHD) is a neurodevelopmental disorder characterized by developmentally inappropriate and impairing patterns of inattention, hyperactivity, and impulsivity across settings [1]. ADHD can interfere substantially with academic performance, peer relationships, family functioning, and emotional well-being. Diagnosis requires evidence that symptoms are not better explained by another mental, developmental, or medical condition and that the associated impairment occurs in more than one setting [1,2].

ADHD in girls may be underrecognized because girls, on average, are more likely to present with inattentive symptoms, internalizing difficulties, or compensatory strategies and may display fewer disruptive behaviors than boys [3,4]. Consequently, some girls do not receive a formal evaluation until increasing academic and interpersonal demands exceed their ability to compensate. Nevertheless, girls can also present with prominent hyperactivity, impulsivity, emotional dysregulation, and interpersonal conflict, as illustrated by the present case.

Accurate assessment of ADHD requires consideration of developmental, behavioral, educational, family, and psychosocial factors that may influence symptom expression, functional impairment, and treatment engagement. Difficulties within the home environment, interpersonal conflicts, school-related stressors, and other contextual influences may complicate presentation without fully accounting for the core ADHD syndrome. Understanding these factors is essential to developing a comprehensive formulation and individualized treatment plan.

Developmental and psychosocial factors may complicate assessment without necessarily accounting for the core ADHD syndrome. Prematurity, for example, has been associated with an increased likelihood of later neurodevelopmental and behavioral difficulties, including ADHD, although this association should not be interpreted as establishing causation in an individual patient [5]. Family conflict may also intensify impairment related to impulsivity and emotional dysregulation and influence how symptoms are understood and managed by caregivers. Educational challenges and school-based difficulties may further contribute to symptom burden and functional impairment, underscoring the importance of obtaining information across settings when evaluating children for ADHD.

Research has additionally reported an association between neurodevelopmental conditions and gender diversity or gender variance in some clinical populations [6,7]. However, gender expression, gender identity, and neurodevelopmental diagnosis are distinct constructs. Gender-nonconforming clothing, activities, or peer preferences do not independently establish gender dysphoria or a gender-diverse identity. Clinicians should therefore approach such expression with developmentally appropriate curiosity and without pathologizing it or assuming that ADHD explains it.

This case was selected because it illustrates the complex interaction between ADHD symptomatology, family relational challenges, educational functioning, and persistent gender-nonconforming expression in a preadolescent child. The report describes the evaluation and treatment of a 10-year-old girl with ADHD, combined presentation, whose clinical course was influenced by family conflict and school-related difficulties within the context of longstanding gender-nonconforming expression. The case emphasizes the value of integrating neurodevelopmental assessment, family-systems evaluation, school-based support, and respectful exploration of the child's developing sense of self to achieve accurate diagnosis and effective intervention.

## Case presentation

A 10-year-old fourth-grade girl living with her mother, father, and twin sister was referred for behavioral evaluation following concerns raised by her school. Teachers reported persistent difficulty sustaining attention, frequent interruption of classroom activities, difficulty waiting her turn, impulsive behavior, academic underperformance, and physical altercations with peers. These concerns had become increasingly disruptive to her educational progress and social functioning.

The patient was born prematurely at approximately 31 weeks of gestation following a complicated delivery and required neonatal intensive care for one month. She experienced minor early motor delays attributed to prematurity, but her subsequent language development was reported to be within expected limits. Her medical history was otherwise unremarkable, with no reported chronic medical illness or significant head trauma.

She had no history of psychiatric treatment or previous formal neurodevelopmental diagnosis and had not received psychotropic medication at the time of the initial assessment. The family psychiatric history was notable for ADHD in a paternal uncle and generalized anxiety disorder in her mother (Table 1).

**Table 1:** Patient Demographic and Clinical Characteristics. ADHD: attention-deficit/hyperactivity disorder

Variable	Findings
Age	10 years old
Sex Assigned at Birth	Female
Grade	4th grade
Living Situation	Resides with mother, father, and twin sister
Birth History	Premature birth at 31 weeks gestation
NICU Stay	1 month
Medical History	Prematurity; otherwise, unremarkable
Psychiatric History	No previous psychiatric diagnosis or treatment
Medication History	No prior psychotropic medication use
Family Psychiatric History	Paternal uncle with ADHD; maternal anxiety
Referral Source	School referral
Primary Concerns	Inattention, impulsivity, aggression, academic difficulties
Final Diagnosis	ADHD, combined presentation

The evaluation incorporated a developmental and psychiatric history, information from the patient and caregivers, school reports, behavioral observations, and assessment of symptoms across home and school settings. The patient demonstrated clinically significant symptoms in both the inattentive and hyperactive-impulsive domains, with associated academic, social, and family impairment (Table 2). The pattern, duration, and cross-setting nature of her symptoms supported a DSM-5-TR (Diagnostic and Statistical Manual of Mental Disorders, Fifth Edition Text Revision) diagnosis of ADHD, combined presentation [1].

**Table 2:** DSM-5-TR ADHD Symptoms Identified During Evaluation [1,2]. DSM-5-TR: Diagnostic and Statistical Manual of Mental Disorders, Fifth Edition Text Revision; ADHD: attention-deficit/hyperactivity disorder

Symptom Domain	Clinical Findings
Inattention	Difficulty sustaining attention
Inattention	Easily distracted
Inattention	Incomplete schoolwork
Inattention	Organizational difficulties
Hyperactivity	Restlessness
Hyperactivity	Frequent movement and fidgeting
Hyperactivity	Difficulty remaining seated
Impulsivity	Interrupting activities
Impulsivity	Difficulty waiting turn
Impulsivity	Aggressive peer interactions
Functional Impairment	Academic problems
Functional Impairment	Family conflict
Functional Impairment	Peer difficulties

Specific learning disorder, oppositional defiant disorder, and autism spectrum disorder were considered in the differential diagnosis. Available educational and clinical information did not support a specific learning disorder. Although the patient displayed argumentative and aggressive behavior, these difficulties occurred primarily in association with impulsivity, frustration, emotional dysregulation, and inconsistent family responses; a separate diagnosis of oppositional defiant disorder was therefore not assigned. Autism spectrum disorder was also considered, but the comprehensive evaluation did not identify the persistent social-communication deficits and restricted or repetitive patterns required for that diagnosis. Continued developmental monitoring was recommended because symptoms and functional demands can evolve over time (Table 3).

**Table 3:** Differential Diagnostic Considerations [1,2]. ADHD: attention-deficit/hyperactivity disorder

Disorder Considered	Findings	Outcome
ADHD	Symptoms present across multiple settings	Confirmed
Autism Spectrum Disorder	No persistent social-communication deficits or restricted interests	Not supported
Oppositional Defiant Disorder	Behaviors related primarily to impulsivity and emotional dysregulation	Not diagnosed
Specific Learning Disorder	Academic difficulties did not suggest a discrete learning disability	Not diagnosed
Anxiety Disorders	Anxiety symptoms insufficient to explain presentation	Not primary diagnosis

During the mental status examination, the patient appeared her stated age and was appropriately groomed. She was restless, frequently changed posture, fidgeted with objects, and required redirection to remain seated. Eye contact was intermittent but appropriate. Her speech was normal in rate, rhythm, and volume, although she occasionally became tangential when distracted. Her mood was generally euthymic with visible frustration when discussing school and family difficulties. Her affect was reactive and mildly labile and brightened when she described preferred activities.

Her thought process was generally linear and goal-directed but was periodically interrupted by distractibility. There was no evidence of hallucinations, delusions, or suicidal or homicidal ideation. Recent and remote memory were intact, and she was oriented to person, place, time, and situation. Attention and concentration were impaired, particularly during nonpreferred tasks or in the presence of environmental distractions. Her judgment was considered developmentally appropriate but affected by impulsivity. She demonstrated emerging insight into her executive-function difficulties, at one point explaining, "It's not me; it's my brain."

Although the school referral emphasized classroom hyperactivity and impulsivity, the family described equally significant challenges at home. The patient experienced recurrent verbal and physical conflict with her twin sister and frequent tension with her father and grandmothers. Differences among caregivers regarding discipline, expectations, and responses to disruptive behavior appeared to reinforce inconsistent behavioral patterns and heightened emotional reactivity. The twin relationship was especially important because persistent comparison and competition complicated the patient’s development of an individual identity within the family.

The developmental history also revealed a longstanding pattern of gender-nonconforming expression. Beginning at approximately four years of age, the patient preferred clothing, colors, activities, and peer associations that her family and cultural environment traditionally associated with boys. At a recent family wedding, for example, she chose masculine-presenting attire. During the observation period, however, she did not report discomfort with her body or assigned sex, request another name or pronouns, or express a different gender identity. Her preferences were therefore documented as gender-nonconforming expression rather than interpreted as evidence of gender dysphoria. The therapeutic approach remained open, supportive, and nonjudgmental, allowing her to describe her preferences without pressure to adopt or reject any identity label.

Therapeutic management

A multimodal treatment plan was developed to address the patient's ADHD symptoms, emotional dysregulation, academic impairment, and family relational difficulties. Weekly individual psychotherapy focused on age-appropriate psychoeducation about ADHD, emotional awareness, impulse control, problem-solving, and practical executive-function strategies. The therapist helped the patient understand that ADHD could make concentration and self-regulation more difficult while reinforcing that the diagnosis did not define her identity or eliminate personal responsibility. Her statement that "it's my brain" was used therapeutically to validate her neurodevelopmental difficulties while helping her identify behaviors that she could learn to manage.

Behavioral parent training was introduced to improve caregiver consistency and strengthen positive parent-child interactions. Caregivers were encouraged to establish clear expectations, provide brief and specific instructions, use immediate positive reinforcement, and apply predictable consequences. A home-based token system was developed to reinforce targeted behaviors, including completing routines, using nonaggressive communication, and pausing before reacting during conflict. Behavioral parent training is included in evidence-based multimodal approaches to pediatric ADHD, although outcomes may vary across settings and measures [2,8,9].

School-based interventions were coordinated through a Section 504 accommodation plan. Supports included preferential seating, movement breaks, shortened or segmented assignments, reduced environmental distractions, written instructions, and regular communication between caregivers and school personnel. The treatment team emphasized the need for interventions across settings because improvement in one environment does not automatically generalize to another.

Family-based sessions were conducted every two weeks. Parent-child work focused on communication, emotional validation, consistent limit-setting, and reduction of escalating interactions. Sessions involving the patient and her twin sister addressed rivalry, physical aggression, personal boundaries, cooperative problem-solving, and the need for each child to be recognized as an individual. Additional work with the grandmother focused on clarifying adult roles and reducing contradictory responses to the patient’s behavior. The family intervention did not treat family conflict as the cause of ADHD; rather, it addressed relational patterns that could intensify the patient’s impairment or interfere with treatment.

The patient's gender-nonconforming expression was not designated as a symptom requiring correction. Clinicians supported her freedom to discuss clothing, activities, friendships, and self-perception in a safe environment. Caregivers were encouraged to avoid ridicule, coercion, or premature conclusions about her future identity. Ongoing assessment focused on her emotional well-being, exposure to bullying or rejection, family acceptance, and any future distress related to identity or bodily development.

After six months, caregivers and teachers reported fewer physical altercations, improved classroom participation, and greater ability to identify and communicate frustration before escalation. Family members also described modest improvement in consistency and conflict management. Clinically significant attentional difficulties remained, particularly during prolonged or nonpreferred academic tasks. Continued multimodal treatment and reassessment of treatment options were recommended.

## Discussion

This case illustrates how ADHD in a preadolescent girl may become most visible through the interaction of attentional impairment, hyperactivity, impulsivity, emotional dysregulation, academic expectations, and family conflict. Girls with ADHD remain vulnerable to delayed recognition when clinicians, families, or educators expect a predominantly male-typical behavioral profile. Although many girls exhibit less overt hyperactivity, others present with combined symptoms and substantial externalizing behavior. Assessment should therefore focus on the individual child’s developmental history, cross-setting symptoms, and functional impairment rather than relying on gender-based assumptions [3,4].

The patient’s premature birth represented a relevant developmental consideration but did not establish the etiology of her ADHD. Large cohort research has found an association between preterm birth and subsequent ADHD, with risk generally increasing at earlier gestational ages [5]. Her family history of ADHD also supported the possibility of inherited vulnerability. The case should consequently be understood through a multifactorial framework rather than attributed to any single perinatal, genetic, or psychosocial factor.

The patient’s emerging insight was clinically meaningful. Her statement, "It's not me; it's my brain," may be interpreted as an early attempt to distinguish her sense of self from the cognitive and behavioral difficulties she experienced. Such language can support psychoeducation and reduce shame, but it must be handled carefully. Clinicians can validate that ADHD affects executive control while teaching that neurodevelopmental vulnerability and behavioral accountability can coexist.

Family-systems considerations

ADHD affects not only the diagnosed child but also the family relationships surrounding the child. Repeated forgetfulness, interruption, impulsive aggression, and emotional escalation may evoke frustration and inconsistent responses from caregivers. In turn, unpredictable discipline, criticism, sibling comparison, and intergenerational disagreement may exacerbate conflict and reduce treatment adherence. The goal of a family-systems approach is not to assign blame or describe the family as the origin of ADHD. Instead, it identifies relational patterns that can either buffer or magnify the child’s impairment.

The twin relationship added complexity. Twin siblings may experience frequent comparison, competition for attention, and difficulty establishing separate identities. In this case, targeted sibling sessions provided opportunities to address aggression and rivalry while helping both children develop clearer boundaries and individual roles. Including extended family members was also clinically relevant because the grandmothers participated in caregiving and behavioral decision-making (Table 4).

**Table 4:** Family-Systems Assessment [8,9].

Family Relationship	Clinical Observation
Mother-Patient	Frequent behavioral struggles but strong support
Father-Patient	High-conflict interactions
Twin Sister-Patient	Rivalry and physical/verbal conflict
Grandmother-Patient	Boundary and discipline disagreements
Overall Family System	Inconsistent behavioral expectations and responses

ADHD and gender expression

The patient’s longstanding gender-nonconforming expression required careful, respectful assessment, but it was not evidence of psychopathology. Gender expression refers to outward presentation, including clothing, interests, mannerisms, and social preferences, whereas gender identity refers to a person’s internal sense of gender. Gender dysphoria involves clinically significant distress or impairment associated with incongruence and should not be inferred from clothing or activity preferences alone [1].

Some studies have identified higher rates of reported gender variance or gender diversity among neurodevelopmental populations, including those with ADHD and autism spectrum disorder [6,7]. These findings describe associations at the group level and do not demonstrate that neurodevelopmental conditions cause gender diversity. They also do not permit clinicians to predict a particular child’s future identity. In the present case, the appropriate response was to document the patient’s expression, assess for distress or impairment, and create a space in which her self-understanding could develop without coercion or premature labeling (Table 5).

**Table 5:** Gender Expression Assessment [6,7].

Domain	Findings
Childhood Expression	Preference for traditionally masculine clothing and activities
Peer Preferences	Greater comfort with male peer groups
Gender Identity Concerns	None reported
Body Dissatisfaction	None reported
Pronoun Change Request	None
Gender Dysphoria	Not identified
Clinical Approach	Affirmative and nonjudgmental monitoring

Therapeutic implications

The observed improvement after six months supports the practicality of multimodal care, but causal conclusions cannot be drawn from a single case (Table 6). Behavioral parent training, school accommodations, psychotherapy, and family intervention were implemented simultaneously, making it impossible to isolate the contribution of any one component (Table 7). Reports of progress were also based primarily on caregiver and teacher observations rather than standardized repeated outcome measures.

**Table 6:** Multimodal Treatment Plan [2,8,9]. ADHD: attention-deficit/hyperactivity disorder

Intervention	Frequency	Goal
Individual Psychotherapy	Weekly	Executive functioning and emotional regulation
Behavioral Parent Training	Ongoing	Improve consistency and reinforcement
School Accommodation (504 Plan)	Continuous	Academic success
Family Therapy	Every 2 weeks	Reduce family conflict
Sibling Sessions	Every 2 weeks	Address rivalry and aggression
Psychoeducation	Ongoing	Increase ADHD awareness

**Table 7:** Clinical Outcomes After Six Months [2,8,9].

Outcome Domain	Baseline	Six Months
Classroom Participation	Poor	Improved
Aggressive Incidents	Frequent	Reduced
Emotional Regulation	Limited	Improved
Parent-Child Conflict	High	Moderate
Sibling Conflict	Severe	Improved
Sibling Conflict	Severe	Severe
Attention Difficulties	Severe	Persistent
Academic Functioning	Impaired	Mild Improvement

Future clinical management should include longitudinal monitoring of ADHD symptoms, academic performance, interpersonal functioning, and emotional well-being. Reassessment may be necessary if attentional impairment remains functionally significant despite consistent psychosocial and educational interventions. Any discussion of additional treatment should follow shared decision-making among the patient, caregivers, and qualified clinicians and should consider symptom severity, functional impairment, preferences, and the overall risk-benefit profile.

This report is further limited by its presentation of a single patient and the absence of long-term follow-up. Family composition, cultural expectations, school resources, and access to treatment may limit generalizability. Moreover, the coexistence of ADHD, family conflict, and gender-nonconforming expression should not be interpreted as proving a causal relationship among these factors.

## Conclusions

This case demonstrates the value of assessing pediatric ADHD within the child’s developmental, educational, and family contexts. Treatment that combined individual psychotherapy, behavioral parent training, school accommodations, and family-based intervention was associated with fewer aggressive incidents, improved classroom participation, and better emotional regulation after six months, although attentional symptoms persisted.

The case also emphasizes that gender-nonconforming expression should not be pathologized or presumed to result from ADHD. Clinicians should distinguish gender expression from gender identity, assess the child’s experience without imposing labels, and support development in a safe and nonjudgmental environment. Such an approach can address neurodevelopmental impairment while respecting the child’s individuality and evolving self-understanding.
